# Decreased defluorination using the novel beta-cell imaging agent [^18^F]FE-DTBZ-d4 in pigs examined by PET

**DOI:** 10.1186/2191-219X-1-33

**Published:** 2011-12-05

**Authors:** Mahabuba Jahan, Olof Eriksson, Peter Johnström, Olle Korsgren, Anders Sundin, Lars Johansson, Christer Halldin

**Affiliations:** 1 Karolinska Institutet, Department of Clinical Neuroscience, Centre for Psychiatry Research, Building R5:U1, Karolinska University Hospital, SE 171 76, Stockholm, Sweden; 2 Department of Radiology, Oncology and Radiation Sciences, Division of Radiology, Uppsala University, SE 751 87 Uppsala, Sweden; 3 Department of Radiology, Karolinska University Hospital, Stockholm, 171 76, Sweden; 4 AstraZeneca R&D, SE 151 36 Södertälje, Sweden; 5 Department of Immunology, Genetics and Pathology, Division of Immunology, Uppsala University, SE 751 87 Uppsala, Sweden; 6 AstraZeneca R&D, SE 431 50 Mölndal, Sweden

**Keywords:** beta cell mass, dihydrotetrabenazine, PET, VMAT2

## Abstract

**Background:**

Fluorine-18 dihydrotetrabenazine [DTBZ] analogues, which selectively target the vesicular monoamine transporter 2 [VMAT2], have been extensively studied for *in vivo *quantification of beta cell mass by positron-emission tomography [PET]. This study describes a novel deuterated radioligand [^18^F]fluoroethyl [FE]-DTBZ-d4, aimed to increase the stability against *in vivo *defluorination previously observed for [^18^F]FE-DTBZ.

**Methods:**

[^18^F]FE-DTBZ-d4 was synthesized by alkylation of 9-*O*-desmethyl-(+)-DTBZ precursor with deuterated [^18^F]FE bromide ([^18^F]FCD_2_CD_2_Br). Radioligand binding potential [BP] was assessed by an *in vitro *saturation homogenate binding assay using human endocrine and exocrine pancreatic tissues. *In vivo *pharmacokinetics and pharmacodynamics [PK/PD] was studied in a porcine model by PET/computed tomography, and the rate of defluorination was quantified by compartmental modeling.

**Results:**

[^18^F]FE-DTBZ-d4 was produced in reproducible good radiochemical yield in 100 ± 20 min. Radiochemical purity of the formulated product was > 98% for up to 5 h with specific radioactivities that ranged from 192 to 529 GBq/μmol at the end of the synthesis. The *in vitro *BP for VMAT2 in the islet tissue was 27.0 ± 8.8, and for the exocrine tissue, 1.7 ± 1.0. The rate of *in vivo *defluorination was decreased significantly (*k*_defluorination _= 0.0016 ± 0.0007 min^-1^) compared to the non-deuterated analogue (*k*_defluorination _= 0.012 ± 0.002 min^-1^), resulting in a six fold increase in half-life stability.

**Conclusions:**

[^18^F]FE-DTBZ-d4 has similar PK and PD properties for VMAT2 imaging as its non-deuterated analogue [^18^F]FE-DTBZ in addition to gaining significantly increased stability against defluorination. [^18^F]FE-DTBZ-d4 is a prime candidate for future preclinical and clinical studies on focal clusters of beta cells, such as in intramuscular islet grafts.

## Introduction

Dihydrotetrabenazine [DTBZ], derived from the neuroactive pharmaceutical tetrabenazine, is a ligand for the vesicular monoamine transporter 2 [VMAT2] which is expressed primarily in cells associated with the regulation of neurotransmission. It is especially associated with the dopaminergic system, and [^11^C]DTBZ has previously been used to study, for example, aspects of dementia [1] and Parkinson's disease [2] in the clinic. High VMAT2 expression has also been found in insulin-producing beta cells in the endocrine islets of Langerhans, but not in the exocrine pancreas [3], and the regional receptor density in the pancreas has therefore been hypothesized to reflect actual beta cell mass [BCM].

Several analogues of DTBZ are currently being explored as putative positron-emission tomography [PET] tracers for the quantification of BCM, including [^11^C]DTBZ [4-6], [^18^F]fluoroethyl [FE]-DTBZ [7], and [^18^F]FP-DTBZ [8-10]. Finding an ideal biomarker to quantify BCM is a big challenge as beta cells are low in abundance (1% to 2%) and dispersed all over the pancreas. [^11^C]DTBZ PET studies in humans have shown initial promising results for the visualization of the pancreas [6]. However, recent studies have shown conflicting results, and there is some concern over the substantial nonspecific uptake of the tracer in the exocrine pancreas [4,7]. Using [^11^C]DTBZ as a lead compound, recently, [^18^F]fluoroalkyl derivatives [11] and other derivatives of DTBZ analogues [12] with improved properties over [^11^C]DTBZ have been developed. Fluorine-18 has longer half-life (109.8 min) which can extend the PET experimental time longer than 100 min and convenient for a long-distance transportation to other sites not possessing cyclotron facilities.

Our group has previously investigated [^18^F]FE-DTBZ as a BCM imaging agent [7]. We found that the *in vitro *binding characteristics of [^18^F]FE-DTBZ in the pure endocrine islet tissue was very favorable, but the relatively high non-displaceable binding to the exocrine pancreatic tissue was still present despite the increased lipophilicity compared to [^11^C]DTBZ. *In vivo *studies in a large animal piglet model revealed highly gradual accumulation of radioactivity in the joints and bone during the PET/computed tomography [PET/CT] examination performed 90 min after the tracer administration. Initially, the accumulation of the tracer in the bone was low and showed a linear increase at the end of the experiments; the spinal column reached a standardized uptake value [SUV] of 3.1. By considering the characteristic of the [^18^F]F^- ^which accumulates readily to the bone [13], [^18^F]FE-DTBZ may be decomposed to [^18^F]F^- ^by *in vivo *defluorination. We concluded that although [^18^F]FE-DTBZ has proper pharmacokinetic and pharmacodynamic [PK/PD] characteristics for the assessment of VMAT2 distribution in tissues with low non-displaceable binding, it is still too blunt as an instrument for pancreatic BCM quantification in its current form.

**Figure 1:**
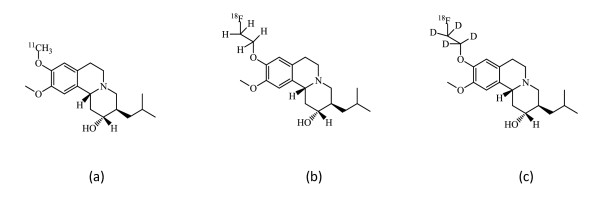
**Structures of [^11^C]-(+)-DTBZ (a), [^18^F]FE-(+)-DTBZ (b), and [^18^F]FE-(+)-DTBZ-d4 (c)**.

In this study, we designed an analogue, [^18^F]FE-DTBZ-d4, with the four ethyl hydrogens of [^18^F]FE-DTBZ substituted with deuterium, as a novel radioligand for quantifying BCM (Figure 1). The hypothesis behind the design of this new radioligand was that the rate of defluorination of the [^18^F]FE moiety would be reduced through a deuterium isotopic effect since the carbon-deuterium [C-D] bond is generally stronger to break than the carbon-hydrogen [C-H] bond. This approach of reducing the rate of defluorination *in vivo *has been previously explored successfully in our group for the synthesis of the norepinephrine transporter radioligand [^18^F]FMeNER-D_2 _[14] and the cannabinoid subtype-1 radioligand [^18^F]FMPEP-d2 [15]. Both of these radiotracers, [^18^F]FMeNER-D_2 _[16] and [^18^F]FMPEP-d2 [17], are used clinically today due to their improved quality by deuteration. Moreover, we can assume that the high affinity and specificity of [^18^F]FE-DTBZ-d4 towards VMAT2 will approximate that of the non-deuterated radioligand [^18^F]FE-DTBZ since the compounds are isosteres.

Here, we report the radiosynthesis of [^18^F]FE-DTBZ-d4 and the measured *in vitro *binding potential [BP] in the pancreas. To test our hypotheses of reduction in defluorination and uptake in the bone while maintaining a similar biological performance (i.e., affinity and specificity to VMAT2), the regional biodistribution of [^18^F]FE-DTBZ-d4 was compared to that of [^18^F]FE-DTBZ and [^18^F]F^- ^in a porcine model by PET/CT.

## Materials and methods

### General

The precursor 9-*O*-desmethyl-(+)-DTBZ [(+)-(2R,3R,11bR)-9-*O*-desmethyl-α-DTBZ] was purchased from ABX GmbH (Radeberg, Germany). The nonradioactive standard FE-(+)-DTBZ-d4 [(2R,3R,11bR)-9-(2-fluoroethoxy)-3-isobutyl-10-methoxy-2,3,4,6,7,11b-hexahydro-1H-pyrido[2,1-a]isoquinolin-2-ol] and the deuterated 2-bromoethyl tosylate (BrCD_2_CD_2_OTs) was purchased from PharmaSynth AS (Tartu, Estonia). All other chemicals were obtained from commercial sources with the highest grade and used without any further purification. Solid-phase extraction [SPE] cartridges, Sep-Pak QMA Light and Sep-Pak tC18 Plus, were purchased from Waters Corporation (Milford, MA, USA). The tC18 Plus cartridge was activated using (1) ethanol [EtOH] (10 mL) and (2) water (10 mL, 18 MΩ). The SPE cartridge Sep-Pak QMA Light was activated using (1) potassium carbonate (K_2_CO_3_) solution (0.5 M, 10 mL) and (2) water (15 mL, 18 MΩ).

### Radiosynthesis of [^18^F]FE-DTBZ-d4 via 2-[^18^F]FE bromide-d4 ([^18^F]FCD_2_CD_2_Br, [^18^F]FEtBr-d4)

#### Production of [^18^F]F^-^

Fluorine-18 fluoride [[^18^F]F*^-^*] was produced from a PETtrace Cyclotron (GEMS, GE, Uppsala, Sweden) using 16.4 MeV protons via the^18^O(p,n)^18^F reaction on^18^O-enriched water [[^18^O]H_2_O]. [^18^F]F*^- ^*was isolated from [^18^O]H_2_O on a preconditioned Sep-Pak QMA Light anion-exchange cartridge and subsequently eluted from the cartridge with a solution of K_2_CO_3 _(1.8 mg, 13 μmol), Kryptofix 2.2.2 (4,7,13,16,21,24-hexaoxa-1,10-diazabicyclo-[8.8.8]hexacosane-K2.2.2) (9.8 mg, 26 μmol) in water (85 μL, 18 MΩ), and MeCN (2 mL) to a reaction vessel (10 mL). The solvents were evaporated at 160°C for 10 to 15 min under continuous nitrogen flow (70 mL/min) to form a dry complex of [^18^F]F^-^/K_2_CO_3_/K2.2.2, and the residue was cooled to room temperature [RT].

#### Radiosynthesis of 2-[^18^F]FE bromide-d4 ([^18^F]FCD_2_CD_2_Br, [^18^F]FEtBr-d4)

2-Bromoethyl tosylate-d4 (15 μL) in *o*-dichlorobenzene [*o*-DCB] (700 μL) was added to the reaction vessel containing dried [^18^F]F^-^/K_2_CO_3_/K2.2.2 complex at RT. The reaction mixture was heated at 135°C for 10 min to produce [^18^F]FEtBr-d4. The crude [^18^F]FEtBr-d4 (boiling point 71.5°C) was purified by distillation at 80°C under nitrogen flow (25 mL/min) and trapped in a second reaction vessel (5 mL) at -15°C containing 9-*O*-desmethyl-(+)-DTBZ precursor (2.0 to 2.5 mg, 6.55 to 8.19 μmol) and NaOH (15 μL, 5 M in water) in anhydrous *N,N*-dimethylformamide [DMF] (500 μL).

#### Radiosynthesis of [^18^F]FE-DTBZ-d4

The reaction mixture containing [^18^F]FEtBr-d4, 9-*O*-desmethyl-(+)-DTBZ precursor, and NaOH in DMF was heated at 110°C for 5 min to produce [^18^F]FE-DTBZ-d4. The crude reaction mixture was diluted with 200 μL water before injecting into a high-performance liquid chromatography [HPLC] semi-preparative reverse-phase μBondapak column (C18, 7.8 Ø × 300 mm, 10 μm, Waters Corporation) for purification. The column outlet was connected to an UV absorbance detector (*λ *= 214 nm) in series with a GM tube for radioactivity detection. Elution with mobile phase CH_3_CN/10 mM H_3_PO_4 _(15:85) at a flow rate of 6 mL/min gave a radioactive fraction corresponding to pure [^18^F]FE-DTBZ-d4 (retention time = 12 min). The fraction was diluted with water (50 mL, 18 MΩ), and the resulting mixture was loaded onto a preconditioned Sep-Pak tC18 Plus cartridge. The cartridge was washed with water (10 mL), and the isolated product, [^18^F]FE-DTBZ-d4, was eluted with 1 mL of EtOH into a sterile vial containing a phosphate-buffered saline solution (7 mL).

### Quality control

The radiochemical purity, identity, and stability of [^18^F]FE-DTBZ-d4 were determined by analytical HPLC using a reverse-phase μBondapak column (C18, 3.9 Ø × 300 mm, 10 μm, Waters Corporation) with mobile phase CH_3_CN/10 mM H_3_PO_4 _(15:85) and a flow rate of 3 mL/min. The effluent was monitored with an UV absorbance detector (*λ *= 214 nm) coupled with a radioactive detector (β-flow, Beckman Coulter, Inc., Fullerton, CA, USA). The identity of [^18^F]FE-DTBZ-d4 was confirmed by co-injection with the authentic nonradioactive FE-DTBZ-d4.

### Specific radioactivity determination

The specific radioactivity [SRA] of the product was measured by the same analytical HPLC method for quality control. SRA was calibrated for UV absorbance (*λ *= 214 nm) response per mass of ligand and calculated as the radioactivity of the radioligand (in gigabecquerels) divided by the amount of the associated carrier substance (in micromoles). Each sample was analyzed three times and compared to a reference standard analyzed three times.

### LC-MS/MS analysis

Liquid chromatography-mass spectrometry [LC-MS/MS] analysis of the purified labeled product, [^18^F]FE-DTBZ-d4, and of the reference standard FE-DTBZ-d4 was performed using a Waters Acquity™ Ultra Performance LC system connected with a Micromass Premier™ Quadrupole Time-of-Flight [TOF] mass spectrometer (Waters Corporation). LC was performed using a Waters Acquity UPLC™ BEH column (C18, 2.1 Ø × 50 mm, 1.7 μm; Waters Corporation) kept at 40°C. The mobile phase consisted of 0.1% formic acid in water (A) and 0.1% formic acid in acetonitrile (B). Samples were analyzed using a linear gradient (0% to 50% B, from 0 to 4 min; 50% to 100% B, from 4 to 4.50 min and then kept at 100% B to 5 min). The flow rate was 0.5 mL/min. The MS was operated in positive-mode electrospray ionization [ESI], with the following settings: capillary voltage 3.0 kV, cone voltage 45 V, source temperature 120°C, dissolvation temperature 350°C, and collision-energy ramp ranging from 20 to 30 eV. [^18^F]FE-DTBZ-d4 was analyzed after radioactive decay without further dilution. FE-DTBZ-d4 (1 mg/mL) was diluted 200 times with water.

### *In vitro *homogenate saturation binding

Endocrine (*n = *4, 80% to 85% islets) or exocrine (*n = *4) tissue preparations were isolated from human pancreata and homogenized in 50 mM tris(hydroxymethyl)aminomethane [TRIS] by a Polytron PT3000 homogenator (Kinematica AG, Littau, Switzerland). The tissue homogenates (0.5 to 6 mg/mL) were incubated in 1 mL of 50 mM TRIS with different concentrations of tracer around an expected dissociation constant *K*_d _of 3 nM. The non-displaceable binding was determined by addition of 10 to 20 μM tetrabenazine (BIOTREND Chemikalien GmbH, Cologne, Germany).

All samples were incubated at RT for 60 min to reach an equilibrium and then moved onto a 1.2 μm Whatman filter (Brandel, Gaithersburg, MD, USA; pretreated with 0.05% polyethylenimine for > 1 h) by a C-48 cell harvester (Brandel, Gaithersburg, MD, USA). The filter components associated to each group were pooled and measured in a well counter (Uppsala Imanet AB, GE Healthcare, Uppsala, Sweden). Tissue samples and references were prepared in triplicates, and filter binding controls, in duplicates.

Tissue protein content (in milligram protein per sample) was assessed by a Bio-Rad Protein Assay (Bio-Rad, Hercules, CA, USA), and absorbance was measured with an EL808 microplate reader (BioTek, Winooski, VT, USA). The BP, defined as the ratio between the receptor density [*B*_max_] and dissociation constant [*K*_d_], was determined by nonlinear regression.

### PET/CT studies

Male Swedish landrace piglets (*n = *4, 15 to 17 kg, 11 to 14 weeks old) were anesthetized by Zoletil forte (Virbac, Carros Cedex, France). The animals were intubated and placed on a ventilator, and the anesthesia was maintained by 2.5% sevofluran. Normoglycemia was confirmed by determining blood glucose content (plasma glucose concentration 4.4 to 7.6 mM). The animal experiments were approved by the local ethics committee for animal research and performed in accordance with local institutional and Swedish national rules and regulations.

The PET/CT studies were performed using a Discovery ST scanner (General Electric, Milwaukee, WI, USA). All scans were acquired in 2D mode and reconstructed by OSEM iterative algorithm. Four piglets were administered 3.4 to 16.3 MBq/kg [^18^F]FE-DTBZ-d4 as an intravenous [i.v.] bolus, and the biodistribution as well as the kinetics of the tracer in the abdomen was studied with a dynamic sequence over 90 min (the head first, prone, 4 frames × 30 s, 3 × 1 min, 5 × 3 min and 14 × 5 min). Arterial blood samples were acquired during the first 90 min, and both the plasma- and whole blood volume-corrected tracer contents were measured using the well counter. A whole-body PET/CT examination was performed 100 to 120 min post-administration.

[^18^F]F^- ^(9.2 to 12.5 MBq/kg) was administered as an i.v. bolus to three piglets to assess the bone uptake PK/PD. Arterial blood and plasma samples were measured using the well-counter. The animals were positioned with a field of view as in the [^18^F]FE-DTBZ-d4 dynamic scan.

The data in this study was compared to previously published PK/PD porcine data on non-deuterated [^18^F]-FE-DTBZ. For details on the methodologies used in this study, see Eriksson et al. [7].

### Image analysis

PET and CT images were analyzed by PMOD (PMOD Technologies Ltd., Zürich, Switzerland). Values are given as means ± standard error of the mean [SEM] unless otherwise stated, and statistics are based on Student's *t *test.

Tissue volumes of interest [VOIs] and regions of interest [ROIs] were delineated on CT images or partially summed PET images. The kinetic uptake in the cortical bone tissue for all three tracers was assessed by generating isocontour VOIs over the lumbar vertrebral bodies in CT images.

### Estimation of tracer defluorination

Since the studies on [^18^F]FE-DTBZ had been performed previously without metabolite correction analysis, we decided to quantify the defluorination retroactively by kinetic modeling of the cortical bone uptake. When modeling the PK/PD of a PET tracer, we generally study the relationship between three different functions (Equation 1) which we can express in a vector form; the input function [*C*_input_], the transfer function [*H*_TR_], and the output function [*C*_tissue_] were defined as follows:

(1) $$C_{\text{in}}⊗H_{\text{TR}}=C_{\text{tissue}}$$

#### Output function

The gradual accumulation of radioactivity in the cortical bone tissue starting around 5 min after administration was assumed to consist entirely of [^18^F]F^- ^uptake. It is difficult to delineate the cortical bone structures in the spinal column due to its relative small thickness even if isocontour thresholding is used. Partial volume effects [PVEs] will still yield kinetics containing contributions from both the cortical bone and bone marrow. Compounding this problem is the observation that the bone marrow expresses VMAT2 and subsequently, will exhibit some degree of specific [^18^F]FE-DTBZ and [^18^F]FE-DTBZ-d4 uptake. To reduce the contribution from non-cortical sources, VOIs over the bone marrow were drawn, and the associated kinetics was subtracted from the cortical isocontour VOIs. The resulting kinetics after this operation was assessed to describe [^18^F]F^- ^PK/PD in the cortical bone tissue and used as the output curve *C*_tissue _for the quantification of tracer defluorination.

#### Transfer function

Cortical bone uptake of [^18^F]F^- ^in pigs is best described by a two-tissue compartment model. The *H*_TR _is given in Equation 2, where *φ*_1_, *φ*_2_, *θ*_1_, and *θ*_2 _are macro-parameters determined by the rate constants *K*_1_, *k*_2_, *k*_3_, and *k*_4 _[18]. *V*_T _and *V*_S _denote the total and specific binding distribution volumes, respectively:

(2) $$H_{TR}=\phi_{1}\times e^{-\theta_{1}\times t}+\phi_{2}\times e^{-\theta_{2}\times t}$$

The [^18^F]F^- ^*H*_TR _in the cortical bone was determined by the kinetic modeling module PKIN for PMOD.

#### Input function

The *C*_input _of [^18^F]F^- ^is in this specific case not created by an instantaneous bolus, but rather from a gradual reaction where [^18^F]FE-DTBZ or [^18^F]FE-DTBZ-d4 is the substrate and [^18^F]F^- ^is the product. The rate of defluorination can then be expressed in a single parameter *k*_defluorination _if we assume that the reaction is concentration-dependent and unchanging over time (or during the time frame investigated here). *C*_in _is then uniquely determined from the arterial blood plasma curve [*C*_p_] and *k*_defluorination _(Equation 3):

(3) $$C_{in}=C_{p}\times e^{-k_{defluorination}\times t}$$

The defluorination rate constant in plasma can then be determined by minimizing Equation 4:

(4) $$NORM\left(C_{tissue}-C_{in}⊗H_{TR}\right),\;where\;C_{in}=C_{p}\times e^{-k_{defluorination}\times t}\geq 0$$

All optimization was performed using MatLab 7.8.0 (MathWorks, Inc., Natick, MA, USA).

### Quantification of pancreatic uptake by kinetic modeling

A one-tissue compartment model was used to estimate the compound parameter *V*_T _as well as the tissue specific rate constants *K*_1 _to *k*_2 _in the pancreas. The *C*_p _was used as an input function, corrected for tracer metabolism by defluorination parameter *k*_defluorination _determined from cortical bone uptake. The kinetic parameters were estimated by the PMOD modeling module PKIN.

## Results

### Radiochemistry

**Figure 2:**
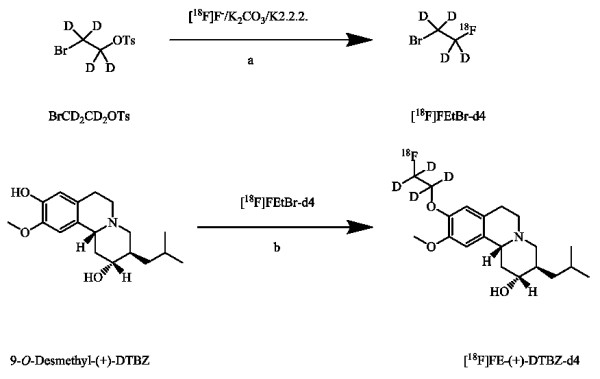
**Synthesis of [^18^F]FE-DTBZ-d4 via [^18^F]FEtBr-d4**. (**a**) *o*-DCB, 135°C, 10 min. (**b**) NaOH, DMF, 110°C, 5 min.

**Figure 3:**
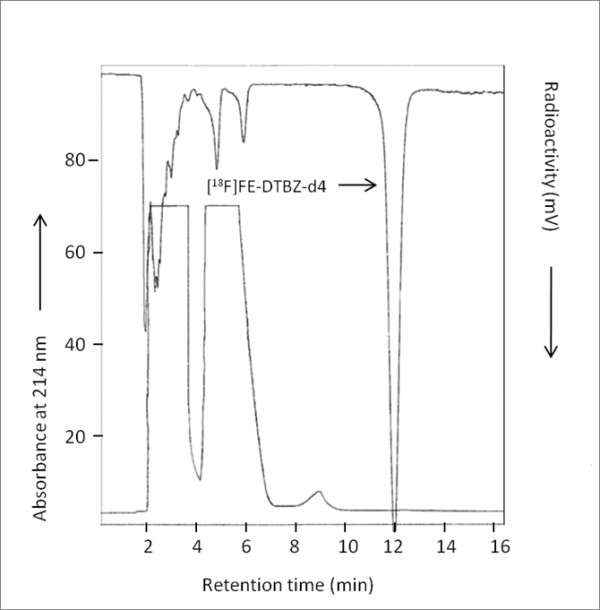
**Chromatogram from the reversed phase semi-preparative HPLC purification of [^18^F]FE-DTBZ-d4**. The upper trace shows radioactivity response, and the lower trace, absorbance response. The mobile phase system is CH_3_CN/10 mM of H_3_PO_4 _(15:85, *v*/*v*) at a flow rate of 6 mL/min.

The radiosynthesis of [^18^F]FE-DTBZ-d4 was successfully performed in two steps and based on the method by Zhang et al. [19]. In the first step, [^18^F]FEtBr-d4 was prepared by a nucleophilic substitution of BrCD_2_CD_2_OTs with [^18^F]F^- ^in the presence of K_2_CO_3 _and Kryptofix 2.2.2. The formed [^18^F]FEtBr-d4 was isolated by distillation and trapped directly in the DMF solution containing precursor 9-*O*-desmethyl-(+)-DTBZ and aqueous NaOH. The purification of [^18^F]FEtBr-d4 by distillation was effective since any nonvolatile impurities were not co-distilling. In the subsequent step, [^18^F]FE-DTBZ-d4 was rapidly formed by [^18^F]fluoroalkylation of the desmethyl precursor. Radiochemical conversion in the second step was approximately 70% (Figure 2). Semi-preparative HPLC purification (Figure 3) and SPE isolation of [^18^F]FE-DTBZ-d4 gave a radiochemically pure compound (> 98%). [^18^F]FE-DTBZ-d4 was produced in good and reproducible radiochemical yield; 1.7 to 3.0 GBq of the pure product was obtained at a beam current of 35 μA and a 20 to 25 min proton bombardment in 100 ± 20 min. The specific radioactivities ranged from 192 to 529 GBq/μmol at the end of the synthesis, and the formulated product was radiochemically stable for up to 5 h (purity > 98%).

### LC-MS/MS analysis

To further identify the product, the ion spectrum of the carrier of [^18^F]FE-DTBZ-d4 was compared to that of the reference compound FE-DTBZ-d4 using LC-ESI-MS/MS-TOF. The ion spectrum of the labeled product was identical to that of the reference compound FE-DTBZ-d4. The labeled product [M+1]^+ ^has a mass-to-charge ratio [m/z] of 356.24 with fragment ions (m/z) 338.23, 310.20, 286.18, and 201.12, and the reference FE-DTBZ-d4 [M+1]^+ ^has a m/z of 356.25 with fragment ions (m/z) 338.23, 310.20, 286.17, and 201.12.

### *In vitro *homogenate saturation binding

The BPs (in femtomoles per milligram protein per nanoMolar) in human pancreatic tissues as determined with [^18^F]FE-DTBZ-d4 are presented in Table 1. The BP was higher in the pure islets (BP_islet _= 27.0 ± 8.8) compared to the exocrine homogenates (BP_exocrine _= 1.7 ± 1.0). The absolute BP was lower in either tissue compared to the non-deuterated FE-DTBZ analogue described previously (BP_islet _= 110.4, BP_exocrine _= 9.8) [7]. However, the BP_islet_/BP_exocrine _ratio was in the same range (16.0 vs. 11.2) as the non-deuterated analogue.

**Table 1:** Total receptor BP determined in either endocrine or exocrine tissue homogenates (*n *= 4)

BP = *B*_max_/*K*_d_^a^		
Tissue batch	Islet	Exocrine
1	29.9	1.0
2	50.1	0.9
3	19.0	0.3
4	9.1	4.5
Average	27.9	1.7
SEM	8.8	1.0

^a^BP was significantly different in the islet and exocrine homogenates (islet/exocrine = 16.0, *p *< 0.05). For reference, the BP values for the non-deuterated analogue in the islet and exocrine homogenates were 110.4 and 9.8, respectively. BP, binding potential; SEM, standard error of the mean.

### [^18^F]FE-DTBZ-d4 biodistribution

**Figure 4:**
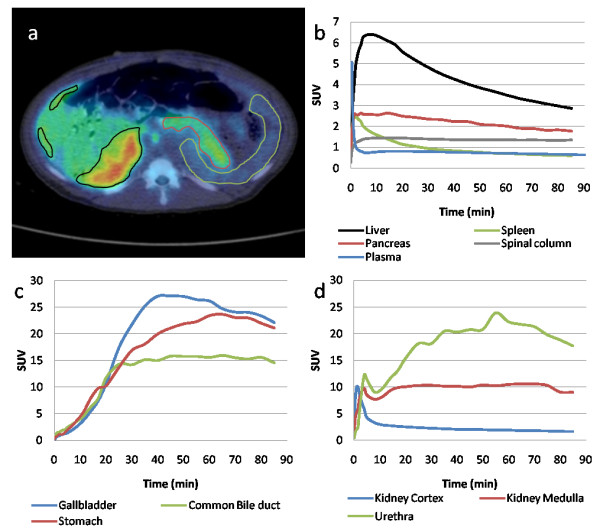
**Transaxial PET/CT fusion image, average dynamic uptake of [^18^F]FE-DTBZ-d4, and tracer excretion**. Delineation of the pancreas (red), spleen (green), and parts of the anterior and posterior hepatic segments (black) exemplified on a transaxial PET/CT fusion image (**a**). The average dynamic uptake of [^18^F]FE-DTBZ-d4 in the pancreas and other abdominal tissues from four different piglets (**b**). Excretion through the biliary system is the fate of a majority of the tracer and its metabolites (**c**), but there is also an elimination of the tracer by urine (**d**).

Accumulation in the pancreas was homogenous with no difference in uptake in the head, body, or tail in either of the subjects with an average peak uptake of SUV 2.64 shortly after administration, falling to SUV 1.8 after 90 min (Figure 4a, b). All piglets were normoglycemic shortly after the induction of anesthesia. Other abdominal tissues with notable accumulation were the liver and spleen, with faster washout kinetics than the pancreas. Parts of the hepatic tracer uptake could be attributed to accumulation in the bile ducts, which drains into the common bile duct (choledoccus) for transport to the gallbladder or the duodenum (Figure 4c). The drainage into the duodenum is transported via the pancreatic duct, which potentially could lead to an overestimation of the islet and/or exocrine uptake. Since no late (30 to 90 min) accretion was seen in the pancreas and no release into the duodenum was detected, it is likely that the pancreatic route of excretion was closed during the studied time frame. The bile, containing high amounts (SUV > 10) of tracer and metabolites, was instead transported into the gallbladder by the cystic duct. In two cases, significant amounts of tracer leaked into the stomach juice (SUV 15 to 20 after 20 min). Apart from the biological elimination through the bile system (usually mainly lipophilic), excretion through the kidneys, urethra, and bladder was observed (Figure 4d).

**Figure 5:**
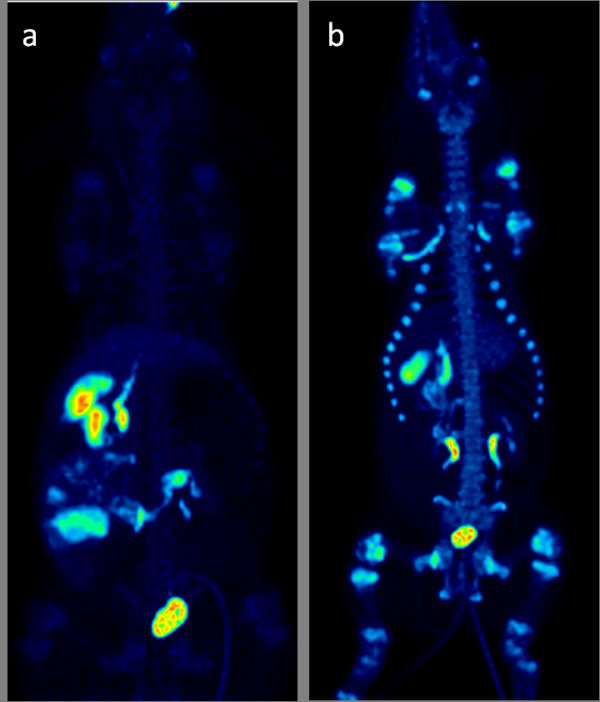
**Three-dimensional maximum intensity projection**. The figure shows 3-D maximum intensity projection 90 min after administration of (**a**) [^18^F]FE-DTBZ-d4; low accumulation in bone structures indicates low levels of free [^18^F]F^- ^and (**b**) non-deuterated [^18^F]FE-DTBZ; high accumulation in bone structures is due to higher levels of free [^18^F]F^-^. Colors indicate SUV 0 (black) to SUV 30 (white).

The deuteration of the tracer was designed to increase the stability to defluorination. The spinal column vertebrae VOIs (containing mainly the cortical bone, but also the trabecular bone tissue due to PVE) showed moderate uptake of tracer (SUV 1.4) within the initial minutes, but no further accumulation (Figures 4b and 5a) indicating low defluorination.

### Tracer defluorination of DTBZ analogues

The two-tissue compartment *H*_TR _for [^18^F]F^- ^into the cortical spinal bone was consistent over all three piglets, predicting large accumulation in the specific binding compartment (*k*_3 _= 0.42 ± 0.04 min^-1^) with close to irreversible kinetics (*k*_4 _= 0.0058 ± 0.0004 min^-1^; Table 2). Almost the entire tissue uptake is predicted to be attributed to the specific compartment (*V*_S_/*V*_T _= 0.99), with less than 1% of the signal coming from the nonspecific or free tracer. The parameter value ratios are comparable to those found previously in a porcine model [20].

**Table 2:** Best fit parameters for describing the *H*_TR _for [^18^F]F^- ^PK/PD in the cortical spinal bone

Parameter	Unit	Pig 1	Pig 2	Pig 3	Average	SEM
*K*_1_^a^	(cc/min)/cc	0.71	0.44	0.56	0.57	0.08
*k*_2_^a^	1/min	1.27	1.78	1.57	1.54	0.15
*k*_3_^a^	1/min	0.39	0.5	0.38	0.42	0.04
*k*_4_^a^	1/min	0.0061	0.0062	0.0051	0.0058	0.0004
*V*_S_^b^	cc/cc	35.9	19.8	26.9	27.53	4.66
*V*_T_^b^	cc/cc	36.4	20.1	27.2	27.9	4.72
*V*_S_/*V*_T_		0.99	0.99	0.99	0.99	0
Plasma delay	s	25.6	23.5	27.9	25.67	1.27
Chi-square fit		0.43	0.41	0.29	0.38	0.04

^a^Represent the rate constants describing tracer flux between the blood plasma and the free/nonspecific tissue compartment (*K*_1 _and *k*_2_) as well as the flux into and out of the specific binding compartment (*k*_3 _and *k*_4_).^b^The distribution volumes of the different compartments are expressed by *V*_s _(specific bound tracer) and *V*_T _(specific bound, nonspecific bound, and free tracers). SEM, standard error of the mean.

Translating the rate constants into biological terms yields a consistent image where the fluoride is distributed into the bone tissue by regular blood perfusion and is incorporated in the cortical bone through mineralization by binding to apatite.

**Figure 6:**
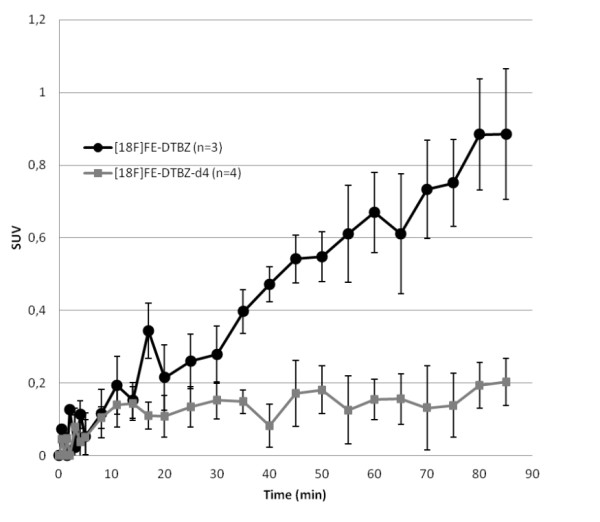
**Uptake of the respective DTBZ analogue in the cortical bone tissue**. The pure cortical uptake was estimated by reducing the influence of partial volume effects due to the non-cortical bone tissue and vascular contributions. This was done by subtracting the trabecular bone uptake from the total uptake in cortical VOIs in the body of the thoracic and lumbar vertebrae in the lower spinal column.

The gradual dynamic defluorination of [^18^F]FE-DTBZ-d4 and [^18^F]FE-DTBZ in plasma was retrospectively determined from the dynamic cortical bone uptake (Figure 6), and the cortical *H*_TR _was acquired from [^18^F]F^- ^data. The defluorination rate constant *k*_defluorination _(Table 3) was significantly lower for the deuterated [^18^F]FE-DTBZ-d4 (*k*_defluorination _= 0.0016 ± 0.0007 min^-1^) compared to the non-deuterated [^18^F]FE-DTBZ (*k*_defluorination _= 0.012 ± 0.002 min^-1^; *p *< 0.05). Alternatively, the defluorination can be expressed as the half-life stability which corresponds to over 6 h for the deuterated tracer compared to just over 1 h for the non-deuterated version.

**Table 3:** Defluorination rate in piglets between the deuterated and non-deuterated FE-DTBZ analogues (*p *< 0.05)

Parameter	Unit	Exp 1	Exp 2	Exp 3		Average	SEM
[^18^F]FE-DTBZ							
*k*_defluorination_^a^	1/min	0.015	0.0079	0.01214		0.012	0.002
*t*_1/2_	min	46.2	87.7	57.1		63.4	
RSS		0.34	1.03	0.65			
[^18^F]FE-DTBZ-d4							
*k*_defluorination_^a^	1/min	0.0016	0.00042	0.0035	0.00088	0.0016	0.0007
*t*_1/2_	min	438.7	1650.4	197.5	787.7	433.9	
RSS		0.50	0.14	0.16	0.16		

^a^The rate constant *k*_defluorination _was almost 7.5 times lower after deuteration (0.0016 ± 0.0007 min^-1 ^vs. 0.012 ± 0.002 min^-1^), resulting in an increased stability half-life (more than 6 h, up from 1 h). *t*_1/2_, half-life; RSS, regression sum of squares; SEM, standard error of the mean.

### Quantification of pancreatic uptake

Table 4 presents the best fit one-tissue compartment model parameters for [^18^F]FE-DTBZ-d4 and [^18^F]FE-DTBZ. The two rate constants were decreased after deuteration (*p*(*K*_1_) = 0.054, *p*(*k*_2_) < 0.05), but the macro-parameter *V*_T _was unchanged (*p *= 0.27). There is no difference in the specific VMAT2 binding in pancreas uptake even though more native [^18^F]FE-DTBZ-d4 is available in the blood plasma. The rate constants represent a higher influx and efflux of the free tracer but have no net accumulation.

**Table 4:** One-tissue compartment model parameters using defluorination-corrected plasma input curves

Parameter	Unit	Exp 1	Exp 2	Exp 3		Average	SEM
[^18^F]FE-DTBZ							
*K*_1_	(cc/min)/cc	0.27	0.30	0.20		0.26	0.03
*k*_2_	1/min	0.08	0.07	0.04		0.064	0.01
*V*_T_^b^	cc/cc	3.39	4.47	4.65		4.17	0.39
Chi-square fit		29.2	19.1	13.3			
[^18^F]FE-DTBZ-d4^a^							
*K*_1_	(cc/min)/cc	0.65	1.09	1.13	0.36	0.81	0.18
*k*_2_	1/min	0.32	0.25	0.37	0.10	0.26	0.06
*V*_T_^b^	cc/cc	2.05	4.39	3.06	3.64	3.69	0.49
Chi-square fit		6.8	4.0	3.7	2.7		

^a^Results for [^18^F]FE-DTBZ-d4 are compared to a reanalysis of [^18^F]FE-DTBZ.^b^The total distribution volume (*V*_T_) is calculated from the rate constant ratio *K*_1_/*k*_2_. *V*_T _is not affected by deuterization even though both *K*_1 _and *k*_2 _are increased. SEM, standard error of the mean.

## Discussion

In this study, we modified the VMAT2 PET ligand [^18^F]FE-DTBZ by deuteration to reduce the rate of defluorination. The fluoroethoxy group of [^18^F]FE-DTBZ can be defluorinated *in vivo *by an initial enzymatic attack at a C-H bond leading to the decomposition of the entire radionuclide carrying the ethyl group. The observed uptake of radioactivity in the cortical bone tissue in the previous study of Eriksson et al.[7] suggested that [^18^F]FE-DTBZ might have been defluorinated extensively *in vivo *by this mechanism. To reduce the rate of defluorination, we investigated if substituting hydrogen for deuterium would increase radioligand stability through a primary isotope effect. It is reported that the cleavage rate of the C-H bond is 6.7 times faster than that of the C-D bond at 25°C [21], and this breakage of the C-H bond is considered to be the rate-determining step in defluorination [22]. There are several examples of PET ligands in the literature where deuterium substitution has improved the *in vivo *metabolic stability [14,15,23,24].

**Figure 7:**
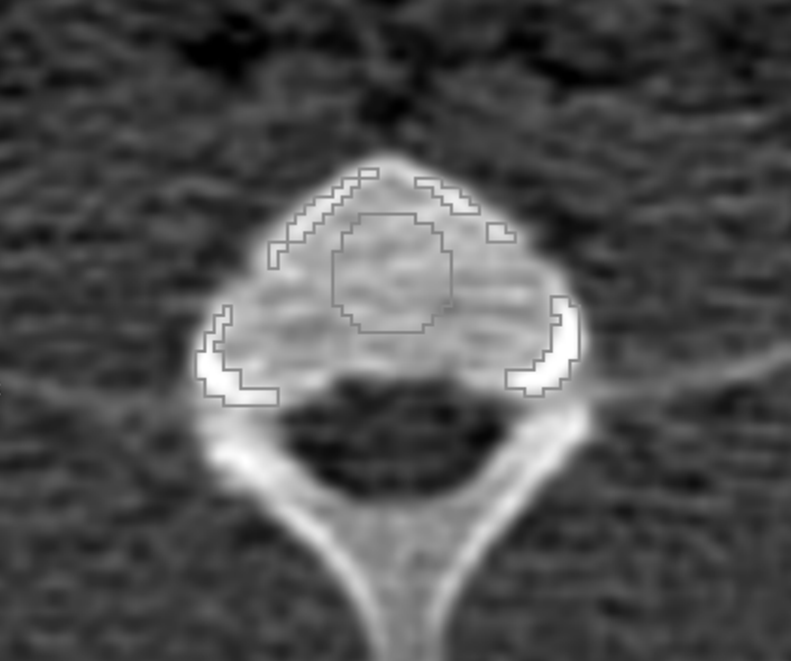
**Computer-assisted delineation of the cortical (white) and trabecular (black) bone tissues in the vertebrae**. Isocontour ROIs were generated by thresholding on CT images. In this example, the cortical and trabecular ROIs have average densities of 391 and 255 HU, respectively.

The main difference in tissue PK/PD between the deuterated and non-deuterated DTBZ analogues occurred in the bone tissue (Figures 5a, b and 6). The bone tissue consists of the cortical bone (the dense bone containing hydroxylapatite) and the trabecular bone (a more porous material containing blood vessels and the bone marrow), and these can be difficult to separate in VOIs, especially in the spinal column where the cortical fraction is relatively low (Figure 7). Fast uptake of the tracer in the vertebrae bone tissue was seen during the first 5 min for both [^18^F]FE-DTBZ-d4 and [^18^F]FE-DTBZ. During the remainder of the scan, there was a close to linear accretion for [^18^F]FE-DTBZ, which was almost completely absent after administration of [^18^F]FE-DTBZ-d4, consistent with the previous work of Schou et al. [14]. We hypothesized that the uptake 'plateau' of SUV 1 to 1.5 for both tracers consisted of both nonspecific binding as well as specific binding to VMAT2 expressed by cells in the bone marrow [25], while the gradual accumulation was due to irreversible [^18^F]F^- ^binding to apatite in the cortical bone. The estimated defluorination rate constant *k*_defluorination _differed significantly between the two deuterated and non-deuterated DTBZ analogues.

PET and/or CT measurements of pancreatic uptake and delineation of the pancreatic tissue contain many potential difficulties, regardless on the animal model studied. Mice and rat models have stretched, diffuse pancreata which can be very difficult to delineate and separate from neighboring tissues, especially without the aid of CT. This is a lesser problem in a large animal model as the pig, which is one of the reasons for the choice of the model in this study. Other difficulties arise from the proximity of high or moderate-uptake regions such as the kidney cortex, the spleen, and potentially, the duodenum. The pancreas can usually be visualized on a few transaxial CT slices (depending on the orientation) without oral contrast administration, but the PET kinetics can still be affected by PVEs (where voxels contain contributions from neighboring tissues). To avoid PVE, we modified the CT-guided pancreatic delineation by studying the kinetics of the neighboring tissues, the kidney, the cortex, and the spleen. The kidney cortex was avoided by studying early-time summations, and the splenic uptake was separable from the pancreatic uptake due to the washout being very rapid during later frames. Further analysis of kinetics using techniques such as masked volume-wise principal compartment analysis could further aid pancreatic delineation.

The tracer-receptor interactions of [^18^F]FE-DTBZ-d4 were seemingly altered by the deuteration although the difference between the two functional groups of O-CD_2_CD_2_F (deuterated) and O-CH_2_CH_2_F (non-deuterated) is too small to change the molecular similarity and bioisoteric properties. However, BP is a composite constant determined by the ratio between *B*_max _and *K*_d_. Therefore, BP can be altered without any change in receptor affinity (*K*_d_). By contrast, *B*_max _can be affected by several factors such as receptor expression in each individual islet/exocrine batch, the availability of receptors due to homogenization, and the determination of the total amount of protein content for individual cell cultures. Most likely, the decreased BP represents differences in islet viability and VMAT2 expression between batches. If the comparison between the tracers were performed on identical islet batches or on a larger group size, we postulate that the difference in the measured BP would decrease. The [^18^F]FE-DTBZ-d4 BP was decreased both in the endocrine and exocrine tissues compared to that of [^18^F]FE-DTBZ. Most importantly, the 16-fold BP_islet_/BP_exocrine _ratio shows that the radiotracer, [^18^F]FE-DTBZ-d4, has similar characteristics for discriminating between the islet and exocrine tissues as the non-deuterated compound, but also a large exocrine nonspecific signal will still be present in the native pancreas *in vivo*.

## Conclusion

In this study, deuterium-substituted [^18^F]FE-DTBZ-d4 was designed, synthesized, and evaluated *in vitro *and *in vivo *as a potential tool for BCM imaging. [^18^F]FE-DTBZ-d4 was synthesized by alkylation of the 9-*O*-desmethyl-(+)-DTBZ precursor via [^18^F]FEtBr-d4 in high radiochemical yield and purity. [^18^F]FE-DTBZ-d4 gained increased stability against defluorination *in vivo *compared to [^18^F]FE-DTBZ and retained a similar BP_islet_/BP_exocrine _ratio. These characteristics are especially important when considering the study of VMAT2 dense tissues in proximity to cortical bone structures, as in the case of intramuscular islet transplantation in preclinical or clinical settings. Although the tracer specificity for beta cells is insufficient for the imaging of endogenous pancreatic islets, it is a prime candidate for future preclinical and clinical studies on focal clusters of beta cells, such as in intramuscular islet grafts. Our results have also demonstrated that the deuterium substitution is an effective tool for improving the properties of the corresponding non-deuterated PET radioligand by reducing their *in vivo *metabolic rates.

## Competing interests

The authors declare that they have no competing interests.

## Authors' contributions

MJ and OE participated in the conception and design of the study, data acquisition, data analysis, data interpretation, drafting and revising the manuscript. PJ, OK, AS, LJ participated in data interpretation and revising the manuscript. CH participated in the conception and design of the study, data analysis, data interpretation, drafting and revising the manuscript. All authors read and approved the final manuscript.
